# Enhancing hospital discharge summary writing skills: a pilot study on a competency-based training model

**DOI:** 10.12688/mep.20928.1

**Published:** 2025-12-17

**Authors:** Marvin Man Ting Chung, Felix Leung, Gabriel Ching Ngai Leung, Anderson Siu Ming Leung

**Affiliations:** 1 Department of Orthopaedics & Traumatology, The University of Hong Kong, Hong Kong, Hong Kong

**Keywords:** Discharge summary, discharge documentation, patient safety, medical writing, medical education

## Abstract

**Background:**

Discharge summary (DS) is an essential clinical document for hospitalized patients. Writing effective DS is a core competency of intern doctors upon entering clinical practice. However, this skill is often underdeveloped due to its exclusion from most medical school curricula, resulting in suboptimal DS quality and communication breakdowns.

**Methods:**

We conducted a one-day, in-person DS writing workshop in June 2025 for 58 final year medical students prior to hospital internship. The workshop comprised of pre-workshop assessments, didactic lectures on DS writing skills, small-group appraisals, and post-workshop assessments with tutor feedback. Pre- and post-workshop surveys assessed participants' perceptions of DS writing and workshop effectiveness using a 5-point Likert scale. DS samples from pre- and post-workshop assessments were graded by three blinded specialist doctors using a 10-component rubric (maximum score: 30, 3 in each component).

**Results:**

98.2% of participants agreed that the workshop improved their DS writing skills and would recommend it to others. Survey reponses showed significant post-workshop improvements in understanding purposes and structure of DS (p<0.001), the requirements for an effective DS (p<0.001), confidence in writing an effective DS (p<0.001), and completing it within 10 minutes (p=0.002). Objective assessment scores also improved significantly across most DS key components and for the total score (20.53 ± 2.86 vs 15.54 ± 3.86, p<0.001).

**Conclusions:**

This is the first report in the literature describing a DS writing educational intervention for orthopedics, as previous reports focused mainly on internal medicine and were not tailored to the specifics of an orthopedic or surgical admission such as procedures, complications, and post-operative instructions. This pilot program improved students’ performance and confidence in DS writing. It highlights the importance in incorporating competency-based, hands-on DS training as a routine component in medical education to better prepare interns for inpatient clinical practice.

## Introduction

Discharge summary (DS) is an essential medical document for all patients after hospitalization and is typically prepared by junior doctors in the public healthcare system. An effective DS conveys essential information about the hospital stay, including diagnosis, clinical progress, complications, treatment details, medication changes, and follow-up instructions. It serves as a vital communication tool between hospital clinicians and outpatient healthcare providers, ensuring continuity of care and promoting patient safety. DSs should also be concise and avoid unnecessary details, so that outpatient clinicians can rapidly retrieve essential information during busy clinics. However, studies indicated that DS quality is often suboptimal, with important information frequently missing or inaccurately documented ^1^.

Intern doctors are required to write DSs from the first day of postgraduate employment. However, for this routine responsibility is rarely included in formal medical school curricula worldwide. Consequently, interns often lack sufficient preparation and experience in generating comprehensive yet concise DSs, which may contribute to medical errors and compromised patient care ^2^. Formal training has been shown to improve patient safety, reduce readmissions, and enhance healthcare system efficiency ^3^. In this context, the primary objective of this pilot study was to address the gap in the local curriculum by providing comprehensive DS training to final year medical students, equipping them with the necessary knowledge and techniques in clinical documentation.

## Methods

### Details of educational intervention

A one-day, in-person DS writing workshop was conducted in June 2025 for 58 final year medical students at the authors’ academic institute. The educational intervention utilized a blended teaching approach, starting with pre-workshop assessments where participants were required to write a DS within 30 minutes using sample anonymized case notes of an orthopedic emergency inpatient admission. Three 15-minute didactic lectures on DS writing principles were then delivered by three orthopedic specialist doctors with the use of slideshow, instructing participants on essential components of DS, suggested techniques and pitfalls to avoid in writing DS.

This was followed by a small-group tutor-led discussion session, during which good, average, and poor anonymized DS samples were provided for group appraisal. Post-workshop assessments were conducted at the end of the training, in the same format as pre-workshop assessment with another set of sample anonymized case notes and the same time constraints. Formative feedback was provided in groups by tutors, who were senior residents and specialist surgeons from the authors' department. Written informed consents were obtained from the participants for participation in the study.

### Evaluation of educational intervention

Pre- and post-workshop surveys were collected from participants. These surveys included various statements on participants’ perceptions of DS writing and the workshop’s effectiveness, rated on a 5-point Likert scale. Participants’ pre- and post-workshop DS sample were collected for assessment via an online learning Moodle platform of the authors’ institution. A total of 57 pre-workshop and 56 post-workshop assessment were collected. Each summary was de-identified and then reviewed by three blinded specialist doctors using a 10-component structured rubric designed by the authors, based on the essential DS components identified before the workshop (Supplementary material 1). The rubric was designed according to a previously published systematic review on the essential components of a good quality DS ^4^, but modified as per the local hospital practice and format. Some of the suggested components were removed from our rubric because components such as admission date, discharge date and discharge medications were automatically generated in the discharge summary by the electronic clinical record system of the hospital, and participants were therefore not required to input the information manually. Each component was graded from 0 (absent information or poorly written) to 3 (excellent and accurate), with a maximum overall total score of 30.

Mean scores of the pre- and post-workshop assessments were calculated from the three independent assessors for quantitative analysis. Variables of the DS samples and survey results were presented as mean ± standard deviation, with comparison between pre- and post-workshop data performed using two-sample t-test after assessment for normality of data. Data analysis was conducted using Stata 18 (StataCorp LLC, College Station, TX, USA). A p-value of <0.05 was considered statistically significant.

## Results

Among the 58 participants with pre-course survey filled, 54 returned the post-course survey. 98.2% of participants agreed that the workshop improved their DS writing skills and would recommend it to others ( Table 1). Most also agreed that learning how to write a DS was an essential skill for interns, whether before or after the workshop. Comparison between pre- and post-workshop surveys showed significant improvement in understanding what constitutes a DS (4.09 0.56 vs 2.28 0.77, p<0.001), the requirements for an effective DS (4.30 0.57 vs 2.53 1.10, p<0.001), confidence in writing an effective DS (3.67 0.67 vs 2.21 0.81, p<0.001) and doing it within 10 minutes (2.74 1.08 vs 2.17 0.80, p=0.002).

**Table 1.** Survey results.

Survey statements	Likert scale (1-5)				
	Mean ± SD			Percentage of score ≥ 4 (%)	
	Pre (n=58)	Post (n=54)	p-value	Pre (n=58)	Post (n=54)
I am familiar with what a discharge summary is.	2.28 ± 0.77	4.09 ± 0.56	**<0.001**	3.5	88.9
Learning how to write a discharge summary is an essential skill for house officers (interns).	4.41 ± 0.99	4.57 ± 0.57	0.30	86.2	96.3
I am confident that I can write an effective discharge summary.	2.21 ± 0.81	3.67 ± 0.67	<0.001	34.5	63
I understand the requirement of an effective discharge summary in a public hospital setting.	2.53 ± 1.10	4.30 ± 0.57	**<0.001**	15.5	94.5
I am confident that I can finish writing a discharge summary within 10 minutes on average.	2.17 ± 0.80	2.74 ± 1.08	0.002	5.2	25.9
Today’s workshop is helpful for improving my discharge summary writing skills.	N/A	4.71 ± 0.47	N/A	N/A	98.2
I recommend this workshop to other medical students.	N/A	4.82 ± 0.39	N/A	N/A	98.2

57 pre-course and 56 post-course DS samples were retrieved for quality evaluation. Assessment scores also improved significantly in most key DS components after the workshop ( Table 2), including primary diagnosis, laboratory data, diagnostic studies or imaging, procedures, secondary diagnosis or other medical problems (p<0.001). The only component of clinical documentation that did not show difference was follow-up plan and instructions (p=0.320). The quality of documentation showed significant improvement after the workshop as well, in terms of comprehensiveness, accuracy, organization and consistency of information, and the appropriate use of terminology and abbreviation (p<0.001). The overall total scores out of 30 were also significantly higher in the post-workshop assessment (20.53 ± 2.86 vs 15.54 ± 3.86, p<0.001). 

**Table 2.** Assessment results.

	Mean ± SD		p-value
	Pre (n=57)	Post (n=56)	
**Components of clinical documentation related to hospitalization**			
Primary diagnosis	1.49 ± 0.36	2.96 ± 0.10	**<0.001**
Laboratory data	1.59 ± 0.67	2.00 ± 0.56	<0.001
Diagnostic studies / imaging	1.43 ± 0.53	1.86 ± 0.45	<0.001
Procedures	1.40 ± 0.57	1.99 ± 0.39	**<0.001**
Secondary diagnosis / other medical problems	1.65 ± 0.61	2.13 ± 0.45	**<0.001**
Follow-up plan and instructions	1.53 ± 0.68	1.65 ± 0.63	0.320
**Overall quality of documentation**			
Comprehensiveness	1.51 ± 0.47	1.90 ± 0.38	**<0.001**
Accuracy	1.47 ± 0.43	1.90 ± 0.42	<0.001
Organization & consistency of information	1.52 ± 0.49	1.96 ± 0.43	**<0.001**
Appropriate use of terminology / abbreviation	1.94 ± 0.38	2.17 ± 0.32	<0.001
**Total**	15.54 ± 3.86	20.53 ± 2.86	**<0.001**

## Discussion

Training initiatives have been shown to enhance the quality of DS significantly ^5^, improving clarity, completeness, and documentation practices ^6^. Programs vary in format, ranging from short one-hour teaching sessions ^7^ to continued feedback throughout a two-week rotation ^8^ or year-long monthly education sessions for residents ^9^. Programs incorporating feedback mechanisms and audits have also been effective in improving DS completeness and quality ^10^. These initiatives not only enhance the quality of DS but also contribute to the well-being of medical students and interns by building their confidence and competence in clinical documentation ^5^, alleviating the pressure faced by junior doctors who often bear the responsibility of writing DS under time constraints. Aligning pedagogical approaches with learners’ preferences and confidence gaps is also critical, to avoid mismatch between educators’ expectations and students’ perceived needs in practical skills acquisition and competency-based training activities ^11^.

The strengths of our educational intervention included its competency-based design, which was tailored to the local medical students. Most previously reported studies have focused on residents ^2, 5, 12^, while few have addressed training outcomes in medical students ^6^. Our workshop adopted a learner-centred approach, incorporating small-group learning and active learning principles. Detailed instructions on DS formats and essentials were provided, as medical students typically have limited knowledge of the documentation process in public hospitals compared to residents. The workshop allowed ample time for skill development and identification of areas for improvement through deliberate practice and formative feedback. Furthermore, the educational intervention was outcome-based, with objective measurement through pre-and post-assessment scores using a structured rubric. Deidentified real-world case notes were used to ensure realistic practice, and the time constraints for writing DS mirrored the working environment of local public hospitals, where all medical graduates begin their internship. The didactic lectures, group appraisal and practice sessions were all catered specifically to the format of DS of the local electronic patient record system, terminology of the local medical system, and the special features of an orthopedic inpatient stay such as details of operations and complications.

As a pilot project, the relatively small sample size was a limitation. Scaling up the project could enhance its generalizability to a wider cohort of medical students. Also, our assessment rubric has not been validated in other studies. However, this was in common with the previously published studies as the format of DS varies significantly among various countries and institutions ^4^, and therefore none of the models were validated externally for systematic evaluation. Future directions include establishing a longitudinal program with interval practices and assessment, where recall of knowledge and sustainable improvement over time could be observed. Structured feedback and rubric-driven practice could also be complemented by gamified formative assessment (e.g. timed DS drafting challenges or leaderboard-supported peer review) to sustain engagement and deliberate practice under realistic time pressure ^13^. Nevertheless, the current study demonstrated its effectiveness and potential for official implementation as an essential training program before the medical students’ transition to formal employment in hospitals. This is also the first report in the literature describing a DS writing educational intervention for orthopedics, as previous reports focused mainly on internal medicine and such studies were not tailored to the specifics of an orthopedic admission such as surgical procedures, complications, and post-operative instructions.

## Conclusion

This was the first DS writing workshop conducted in our locality, addressing a critical gap in medical education, as well as the first DS writing educational intervention described for orthopedics. This pilot program highlights the importance of incorporating competency-based, hands-on training in DS writing as a routine component in medical education to better prepare interns for inpatient clinical practice.

## Ethics approval

The study was approved by the Institutional Review Board of The University of Hong Kong / Hospital Authority Hong Kong West Cluster (reference number: UW 25-372). The study was conducted in accordance with the Declaration of Helsinki.

## Data Availability

Figshare: Discharge summary workshop dataset, https://figshare.com/articles/dataset/Discharge_summary_workshop_dataset/30588491/2 ^14^ This project contains the following underlying data: •    Survey.docx (pre- and post-course questionnaire) •    Survey results 2025 copy.xlsx (pre- and post-course questionnaire responses) •    Assessment score All.csv (pre- and post-course assessment scoring by assessors) •    Subject Information Sheet and Informed Consent.docx (subject information sheet and informed consent for participants) •    Supplementary material 1.docx (discharge summary assessment rubrics) Data are available under the terms of the Creative Commons CC0 license.
