# Supplementary material for: Enhancing hospital discharge summary writing skills: a pilot study on a competency-based training model
Source: MedEdPublish (2016). 2025 Dec 17;15:283. [Version 1] doi: 10.12688/mep.20928.1 (PMC12800595; doi:10.12688/mep.20928.1)
Supplement: Peer review history

# Reviewer report: Reviewer response for version 1

**DOI:** 10.21956/mep.22411.r44929

**Published:** 2026-01-13

**Authors:** Rhoda Meyer

**Affiliations:** 1 Stellenbosch University, Cape Town, Cape Town, South Africa

Thank you for the opportunity to review this report. I found it interesting and relevant for clinical training.

 The authors highlight the importance of writing effective discharge summaries as a core competency for intern doctors. The one-day workshop presented to final year students seems appropriate to develop such a skill. The results reveal that this workshop could be beneficial to students. While this is an interesting study, it could be improved by a focus on both theoretical underpinnings and methodological rigour.

 In the introduction, the authors argue that writing DS is not often taught at the undergraduate level. The lack of effective DS writing could ultimately affect the quality of patient care, especially when information is not accurately documented or missing. While this is an important issue, it needs to be situated within the broader HPE literature to highlight the gap and the relevance of this study to HPE.

 The authors describe a one-day in-person workshop as the intervention for developing effective DS writing. While a good breakdown of the workshop has been provided, the timeframe between the workshop and the assessment is not indicated. The timeframe is important as a more longitudinal approach will help determine whether students have internalised the skill.

 In addition, there is no literature to support the pedagogical or methodological choices made in this pilot study. For example, is there a learning theory that speaks to the way students learn effective writing? Was the choice of the 15-minute didactic lectures and small-group tutorial discussion based on any educational models/frameworks or theories? Why was pre-and post-workshop surveys selected over more qualitative approaches? Was this method of data collection informed by any models, such as Kirkpatrick’s model?

 In terms of ethical considerations, while it is mentioned that the study was approved by the institutional review board of the University of Hong Kong, there is no indication of whether students were given the choice to opt out of the study and if those who opted out still benefited from the workshop. In addition, it’s not clear whether the results were used as part of the students’ assessment. If this were the case, there are a number of ethical aspects that need to be addressed.

 The discussion is well written but requires alignment with the introductory sections. For example, in this section, the authors introduce many new ideas that were not foregrounded in the introduction, such as the value of feedback, building confidence and competence, and matching educators’ expectations with students need.  In certain areas, stronger justifications are needed. For example, it is indicated that the workshop adopted a learner-centred approach, a stronger justification for the use of didactic lectures (which often present students as passive recipients) and how this was adapted to support learner-centeredness is needed.

Have any limitations of the research been acknowledged?

Yes

Is the study design appropriate and does the work have academic merit?

Partly

Is the work clearly and accurately presented and does it cite the current literature?

Partly

If applicable, is the statistical analysis and its interpretation appropriate?

I cannot comment. A qualified statistician is required.

Are all the source data underlying the results available to ensure full reproducibility?

Partly

Are the conclusions drawn adequately supported by the results?

Partly

Are sufficient details of methods and analysis provided to allow replication by others?

Partly

Reviewer Expertise:

health professions education, clinical learning, workplace-based assessment, faculty development

I confirm that I have read this submission and believe that I have an appropriate level of expertise to confirm that it is of an acceptable scientific standard, however I have significant reservations, as outlined above.

# Reviewer report: Reviewer response for version 1

**DOI:** 10.21956/mep.22411.r44928

**Published:** 2025-12-30

**Authors:** Tayseer M Mansour

**Affiliations:** 1 Family & Community & Medical Education, Taibah University Faculty of Medicine (Ringgold ID: 123307), Medina, Al Madinah Province, Saudi Arabia; 2 Medical Education, Suez Canal University Faculty of Medicine (Ringgold ID: 68833), Ismailia, Ismailia Governorate, Egypt

This brief report by Chung et al. addresses the important and often overlooked topic of discharge summary (DS) writing skills among medical students. The authors are to be commended for developing and evaluating a structured, competency-based workshop to address this educational gap. The study is well-structured, with a clear description of the educational intervention and a multi-faceted evaluation approach that includes both subjective participant feedback and objective assessment of DS quality. The finding that a targeted, one-day workshop can significantly improve both students' confidence and their objectively measured performance is a valuable contribution to the medical education literature.

 However, the report has several methodological and reporting limitations that should be addressed to strengthen the validity and impact of the findings. The primary concerns relate to the study design, the statistical analysis, and the reporting of the assessment tool's psychometric properties. The comments below provide specific points for the authors to consider for revision.

 To improve the manuscript, the authors should:

 •Strengthen the introduction with a more detailed literature review to better justify the need for an orthopedic-specific DS writing workshop.

 •More prominently acknowledge the limitations of the single-group study design.

 •Report the inter-rater reliability of the assessment rubric.

 •Justify the use of a two-sample t-test or re-analyze the data with a paired t-test.

 •Provide the full assessment rubric as a supplementary file.

 •Temper the conclusions to reflect the methodological limitations of the study.

Have any limitations of the research been acknowledged?

Yes

Is the study design appropriate and does the work have academic merit?

No

Is the work clearly and accurately presented and does it cite the current literature?

Partly

If applicable, is the statistical analysis and its interpretation appropriate?

Partly

Are all the source data underlying the results available to ensure full reproducibility?

Yes

Are the conclusions drawn adequately supported by the results?

Yes

Are sufficient details of methods and analysis provided to allow replication by others?

Partly

Reviewer Expertise:

Medical Education - I am involved in developing and evaluating educational interventions for medical students, specifically competency-based training programs.

I confirm that I have read this submission and believe that I have an appropriate level of expertise to confirm that it is of an acceptable scientific standard, however I have significant reservations, as outlined above.

# Reviewer report: Reviewer response for version 1

**DOI:** 10.21956/mep.22411.r44922

**Published:** 2025-12-30

**Authors:** Devi Prasad Mohapatra

**Affiliations:** 1 Jawaharlal Institute of Postgraduate Medical Education & Research (JIPMER), Puducherry, India

A discharge summary is a vital clinical document that serves as both a medical and communication tool. From the patient's perspective, it ensures continuity of care by providing clear instructions for follow-up, medication, and warning signs after hospital discharge. For healthcare providers, it plays a critical role in handover communication between hospital-based and community-based practitioners. Improving the quality and clarity of discharge summaries is essential in enhancing overall care coordination.

 The authors have correctly identified that formal training in discharge summary writing is generally missing from the curricula of most of the medical schools.

 The authors have detailed a discharge summary workshop conducted for final year medical students using a blended teaching approach. They gave pre-workshop assessments, allowed them to write discharge summaries and subsequently worked on improving discharge summaries

 The study is well written, and methodology is properly described. It would be better if the authors add the inclusion criteria for the students who participated in the study. The authors note the study's limitation in participant numbers, its focus on orthopedics, and the need for broader research with a larger population.

 Overall, I find the manuscript satisfactory and would recommend authors to carry out this study in a larger population and standardize the workshop methods so that it can be replicated by other institutions.

Have any limitations of the research been acknowledged?

Yes

Is the study design appropriate and does the work have academic merit?

Yes

Is the work clearly and accurately presented and does it cite the current literature?

Yes

If applicable, is the statistical analysis and its interpretation appropriate?

Yes

Are all the source data underlying the results available to ensure full reproducibility?

Yes

Are the conclusions drawn adequately supported by the results?

Yes

Are sufficient details of methods and analysis provided to allow replication by others?

Yes

Reviewer Expertise:

Curriculum Development, Continuing Professional Development, Small Group Teaching. , Digital tools in teaching and learning.

I confirm that I have read this submission and believe that I have an appropriate level of expertise to confirm that it is of an acceptable scientific standard.

# Reviewer report: Reviewer response for version 1

**DOI:** 10.21956/mep.22411.r44921

**Published:** 2025-12-26

**Authors:** Emre Emekli

**Affiliations:** 1 Eskişehir Osmangazi University, Eskişehir Province, Eskişehir Province, Turkey

This study describes a short-term, competency-based educational intervention aimed at improving the writing of discharge summaries (DS). The topic is important in terms of patient safety and clinical communication. However, the originality of the study and its contribution to the literature are limited. The educational design, evaluation approach, and reported outcomes largely overlap with single-group pre–post educational interventions that have been repeatedly described in the literature. In this context, I am of the opinion that the authors have not sufficiently considered the methodological limitations of the existing literature when positioning their study, and that the current work does not offer a new conceptual, methodological, or evaluation-based contribution that goes beyond these limitations.

 Study Design: A single-group pre–post design was used. The absence of a control group makes it impossible to exclude the effects of informal learning and natural time-related development. Although this limitation is well known in the literature, it has not been critically discussed in the study.

 Assessment Tool: The 10-component rubric used was developed by the authors; although it is mentioned as a limitation, no validity or reliability analyses are reported. This constitutes an important methodological gap in terms of the objectivity and reproducibility of the measured outcomes.

 Outcome Level: The evaluation is limited to short-term learning outcomes only. Behavioral change (Kirkpatrick level 3) or reflection on clinical outcomes (level 4) was not assessed. This limits the study’s real contribution to clinical education.

 The authors state that procedures and complications specific to orthopedic admissions are addressed in DS writing. However, this specificity:

 – is not supported by a detailed cognitive task analysis,

 – does not systematically examine typical errors or complexities specific to orthopedic discharge summaries,

 – does not clearly demonstrate how the educational content qualitatively differs from that of other specialties.

 Therefore, the claim of being “orthopedic-specific” remains at the level of a contextual difference rather than representing a pedagogical innovation.

**Other suggestions and critiques:**

**Title**

 While the emphasis on “pilot study” is appropriate, the manuscript claims that the study is orthopedic-specific. Therefore, this should be stated more clearly in the title.

**Introduction**

 The gaps in the literature are clearly presented; however:

 – The reasons why existing DS educational interventions are insufficient, or why an orthopedic-specific approach is needed, could be emphasized more clearly.

 – Challenges specific to orthopedic admissions (surgical procedures, complications, postoperative care) could be positioned earlier in the introduction.

**Methods**

 The internal consistency of the rubric or inter-rater reliability is not reported. At minimum, it is recommended that this issue be explicitly stated as a limitation.

**Statistics**

 A two-sample t-test was used in the statistical analysis; however, given the pre–post design, the rationale for not using a paired analysis (paired t-test) should be more clearly stated, or the reason for this choice should be explained.

**Discussion**

 The discussion emphasizes the strengths of the study; however, it does not sufficiently engage with long-standing methodological issues highlighted in the literature (single-group design, low Kirkpatrick level, short-term assessment). In particular, it is not clearly articulated how the present study differs from previously published similar interventions or which specific gap it fills. A recent systematic review is recommended: *Medical record-keeping educational interventions for medical students and residents: a systematic review* – *Health Information Management Journal*.

**Limitations**

 Due to the short-term nature of the evaluation, the retention of knowledge and skills was not assessed, which constitutes an important limitation.

**In conclusion, this study:**

 – addresses an important educational topic; however,

 – repeats an intervention model that has already been well documented in the literature;

 – does not offer a clear methodological or conceptual innovation that advances the field.

 Therefore, in its current form, the level of original contribution of the study is limited.

Have any limitations of the research been acknowledged?

Yes

Is the study design appropriate and does the work have academic merit?

No

Is the work clearly and accurately presented and does it cite the current literature?

Partly

If applicable, is the statistical analysis and its interpretation appropriate?

Partly

Are all the source data underlying the results available to ensure full reproducibility?

Yes

Are the conclusions drawn adequately supported by the results?

Yes

Are sufficient details of methods and analysis provided to allow replication by others?

Yes

Reviewer Expertise:

Medical education; clinical documentation

I confirm that I have read this submission and believe that I have an appropriate level of expertise to state that I do not consider it to be of an acceptable scientific standard, for reasons outlined above.
